# A Pt(IV) Prodrug Combining Chlorambucil and Cisplatin: A Dual-Acting Weapon for Targeting DNA in Cancer Cells

**DOI:** 10.3390/ijms19123775

**Published:** 2018-11-27

**Authors:** Diego Montagner, Dina Tolan, Emma Andriollo, Valentina Gandin, Cristina Marzano

**Affiliations:** 1 Department of Chemistry, Maynooth University, Maynooth, Ireland; 2 School of Chemistry, National University of Ireland Galway, Galway, Ireland; d_tolan2005@yahoo.com; 3 Department of Chemistry, Faculty of Science, El-Menoufia University, Shebin El-Kom 32511, Egypt; 4 Dipartimento di Scienze del Farmaco, Università degli Studi di Padova, Padova 35131, Italy; emma.andriollo@gmail.com (E.A.); cristina.marzano@unipd.it (C.M.)

**Keywords:** cisplatin, chlorambucil, Pt(IV) prodrugs, cytotoxicity

## Abstract

In this study, two DNA-targeting agents, cisplatin and chlorambucil, were combined in a Pt(IV) prodrug, **1**, which was thoroughly characterized by means of spectroscopic and spectrometric techniques. Tested towards a panel of various human tumor cell lines, this compound showed superior in vitro antitumor potential than the reference drug cisplatin. In addition, an antitumor potential of **1** was found, which is comparable to that of oxaliplatin in 3D spheroid models of colon cancer cells. Mechanistic studies performed in colon cancer cells confirmed that the conjugation of chlorambucil to Pt(IV) cisplatin-based scaffold tunes the lipophilicity of the prodrug, consequently improving the ability of the compound to accumulate into cancer cells and to target DNA, ultimately leading to apoptotic cancer cell death.

## 1. Introduction

**Scheme 1:**
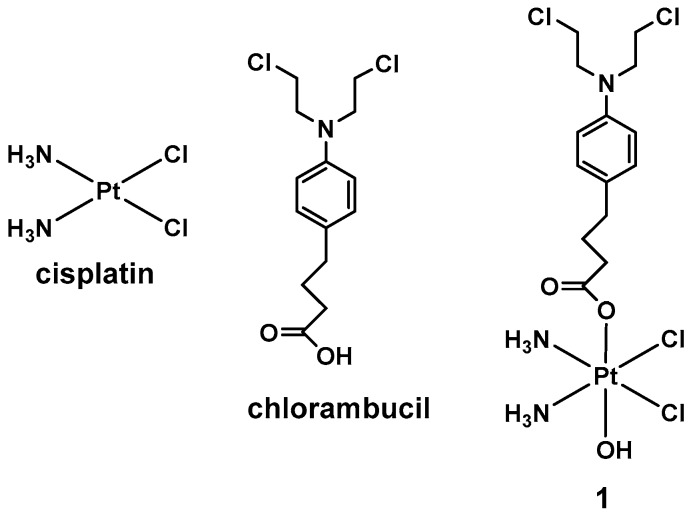
Structure of cisplatin, chlorambucil, and of **1**.

After 40 years since its Food and Drug Administration (FDA) approval as an antitumor agent, cisplatin (CDDP) still today represents one of the most successful drugs worldwide employed for the systemic treatment of solid tumors (Scheme 1) [1,2,3]. Despite its efficacy and success, severe side effects and resistance limit CDDP application in clinic. One of the most promising strategies to overcome the toxicity associated with cisplatin treatment is the use of platinum-based drugs in the oxidation state +4 (Pt(IV)) [4,5,6,7,8]. Pt(IV) are d^6^-octahedral complexes with improved stability compared to the Pt(II) counterparts. This kinetic inertness confers upon them a limited ability to interact with biomolecules, thereby reducing the incidence of undesired toxic side effects and ultimately allowing for oral administration.

Upon cellular internalization, the Pt(IV) species are reduced to the bioactive Pt(II) analogues with the concomitant release of the axial ligands. Therefore, octahedral Pt(IV) complexes offer the opportunity to design multi-acting Pt prodrugs, when the axial ligands are endowed with their own pharmacological activity. Several examples of multi-action Pt(IV) complexes have been reported in literature to date, and the majority of these multi-targeted Pt(IV) derivatives hold axial ligands that target proteins, peptides, and hormones [9,10,11,12,13,14,15,16,17]. On the contrary, only a few Pt(IV) prodrugs containing different types of DNA-damaging drugs as axial ligands have been explored so far.

In this light, we judged it to be of interest to synthetize a Pt(IV) complex based on cisplatin and the alkylating DNA agent chlorambucil. Chlorambucil is a DNA alkylating agent belonging to the nitrogen mustard class of anticancer agents; it can form covalent adducts with the nucleobases of the DNA, alkylating the nucleobases adenine and guanine at the nitrogens N3 and N7 [18,19]. As in the case of cisplatin, DNA cross-linking induced by chlorambucil prevents DNA replication and transcription, leading to cell death (Scheme 1) [20,21,22].

Two recent examples of chlorambucil conjugated with Pt-based drugs have been reported by Dhar and Gou [23,24]. The potent in vitro antitumor activity described for some Pt(IV) complexes combined with chlorambucil suggests that the conjugation of two drugs targeting DNA may be an effective strategy to overcome cisplatin resistance.

Recent publications by our group and others have demonstrated that non-symmetrical Pt(IV) prodrugs (functionalized in only one axial position) are endowed with a better biological activity than their symmetrical counterparts [25,26,27,28]. These behaviors can be explained by considering that the reduction rate plays a prominent role in the biological activity of Pt(IV) drugs. In fact, activation by reduction when both the axial positions are substituted is slower than that of asymmetrical Pt(IV) prodrugs. The presence of an OH group can improve the reduction rate because it can act as a bridging ligand between the metal complex and the reducing agent, allowing for a fast and efficient transfer of electrons to the metal center [29].

On this basis, in this paper we report the synthesis, characterization, and biological investigations concerning a non-symmetrical Pt(IV) prodrug (**1**) based on CDDP, where the two axial ligands are occupied by a chlorambucil molecule and by an OH group, respectively (Scheme 1).

## 2. Results and Discussion

### 2.1. Synthesis and Characterization

**Scheme 2:**
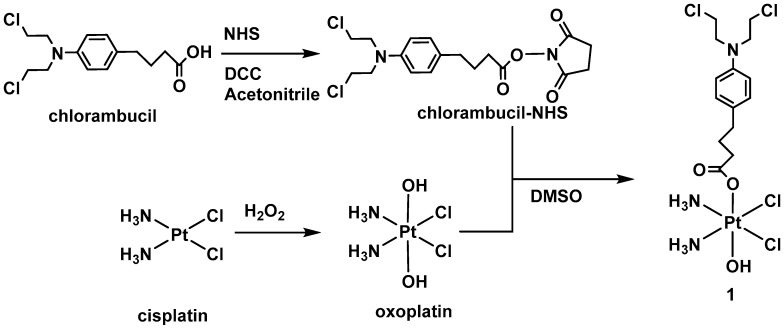
Synthetic pathway for **1**.

The complex presented in this work is a non-symmetrical dual-action Pt(IV) derivative of cisplatin with chlorambucil as a bioactive axial ligand. Pt(IV) complexes are commonly synthetized through the reaction of oxoplatin (Scheme 2) and an activated (anhydride or acyl chloride) carboxylic acid. To avoid bis-substitution, a weaker activator such as NHS ester (N-hydroxo succinimide) is more convenient. **1** was synthetized as described in Scheme 2, by mixing oxoplatin and the chlorambucil-NHS ester (1:0.9 ratio) in dimethylsulfoxide (DMSO).

The final complex was characterized using ^1^H, ^13^C, ^195^Pt NMR, ESI mass spectrometry and elemental analyses (See Figures S1–S6 in the Supplementary Materials). The NH_3_ peak in the ^1^H NMR at 5.93 ppm and the ^195^Pt peak at 994 ppm are in the typical region for mono-substituted Pt(IV) complexes.

**Figure 1:**
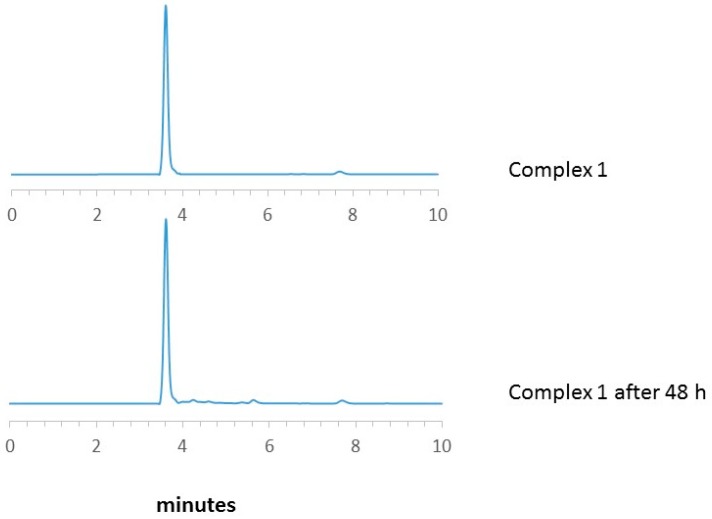
HPLC chromatograms of complex **1** in DMSO/PBS (1/10) at 37 °C.

The stability of **1** was evaluated using an HPLC analysis of a solution of the complex in DMSO/PBS (phosphate buffer saline,1/10) at 37 °C. The chromatogram obtained confirms the purity of the product and its stability for 48 h (Figure 1).

### 2.2. Reduction Studies

Once they have entered cells, Pt(IV) prodrugs are activated through reduction mediated by intracellular reducing agents, such as ascorbic acid and reduced glutathione (GSH), the content of which is much higher in cytoplasm than in extracellular compartments [4].

**Figure 2:**
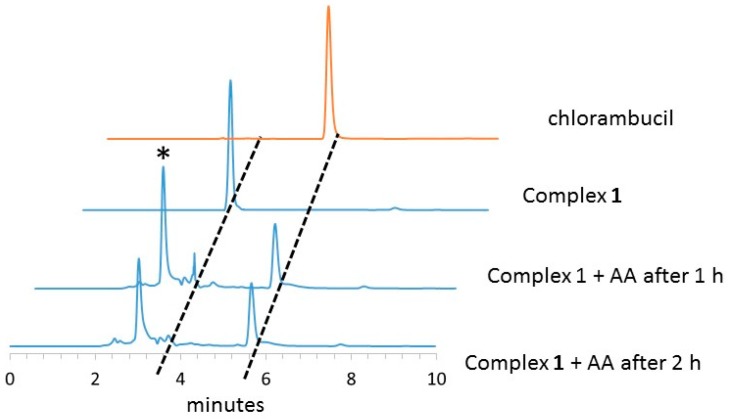
HPLC chromatograms of the reduction of complex **1** by ascorbic acid (AA). (* is AA) (See Materials and Methods for details).

HPLC is a useful tool to study the reduction of Pt(IV) complexes in the presence of an excess of reducing molecules. The rate of reduction was studied by incubating **1** with a ten-fold excess of ascorbic acid. Our results showed that the reaction between the starting Pt(IV) compound and an excess of ascorbic acid leads to the formation of cisplatin with the release of the axial ligand chlorambucil. From a kinetic point of view, **1** is completely reduced after 2 h, as shown in Figure 2.

### 2.3. Cytotoxicity Studies

The dual-action Pt(IV) derivative **1** was screened for antiproliferative activity against a panel of human cancer cell lines using the colorimetric MTT (3-[4,5-dimethylthiazol-2-yl]-2,5 diphenyl tetrazolium bromide) assay. Cell lines representative of colon (HCT-15 and LoVo), pancreatic (BxPC3 and PSN1), ovarian (2008 and C13*), thyroid (BCPAP), and cervical (A431) cancers were treated for 72 h with increasing amounts of **1**, chlorambucil or CDDP, and IC_50_ values were calculated from dose-survival curves (see Table 1).

The reference drugs chlorambucil and CDDP showed a weaker activity compared to the dual-action Pt(IV) prodrug, with IC_50_ concentrations on average one and two orders of magnitude higher than those detected with **1**, respectively. The Pt(IV) prodrug **1** elicited IC_50_ values in the low micromolar range (range 0.28–1.74 µM) against all tested cell lines. In particular, **1** was extremely effective against cancer cells scarcely sensitive to CDDP, such as K-ras mutated highly aggressive PSN1 pancreatic cancer cells and p53 mutated HCT-15 colon cancer cells.

In addition, **1** proved to be equally effective against cisplatin-sensitive (2008) and -resistant (C13*) cancer cells, thus attesting to its ability to overcome cisplatin resistance. Overall, these cytotoxicity results demonstrate, for **1**, a significant increase in potency and a broader spectrum of activity compared to that of the reference Pt(II) parental compound CDDP. Notably, **1** showed on average an in vitro antitumor potential up to ten times higher than those of previously reported chlorambucil-Pt derivatives [23,24].

The in vitro antitumor activity of the Pt(IV) dual-action derivative was also assayed on a 3D cell culture. Cells growing in 3D culture systems form spheroids, which are characterized by cells in various stages. In particular, cells in the outer layers of the spheroid are viable and highly proliferating whereas cells in the core, which receive less oxygen and nutrients, are in a quiescent or hypoxic state [30]. This cellular heterogeneity resembles that of in vivo tumors, making 3D cell spheroid cultures more predictive than conventional 2D monolayer cultures for the screening of putative anticancer drugs.

The growth inhibitory effect was evaluated by means of acid phosphatase (APH) test. Table 2 reports the IC_50_ values obtained on 3D cell spheroids of human HCT-15 colon cancer cells after 72 h of treatment with the Pt(IV) complex **1** as well as CDDP and oxalilplatin (OXP); the latter are used as references.

In colon cancer spheroid cell cultures, (**1**) was found to be significantly more active than CDDP and only slightly less efficient than OXP, a first-line drug in the management of colorectal cancer [31].

### 2.4. Cellular Uptake and DNA Platination

**Figure 3:**
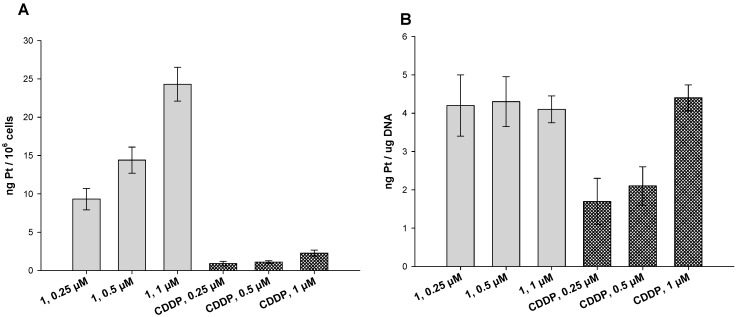
Cellular uptake (**A**) and DNA platination (**B**). (**A**) HCT-15 cells were incubated for 24 h with increasing concentrations of **1** or CDDP. The error bars indicate SD. (**B**) Platination levels of nuclear DNA extracts. HCT-15 cells were treated for 24 h with increasing concentrations of **1** or CDDP. DNA was extracted, quantified, and the amount of Pt bound to DNA was estimated using GF–AAS. Error bars indicate SD.

It has been demonstrated in many reports that the presence of lipophilic ligands in the axial positions can substantially increase the lipophilicity of the whole Pt(IV) cisplatin analogues, thus improving cellular internalization [32,33,34]. This is because drug cellular uptake usually increases with drug lipophilicity, since passive diffusion through cell membrane is facilitated. Therefore, we investigated the cellular uptake as well as the DNA binding of **1** in human colon HCT-15 cancer cells. Cells were treated for 24 h with **1** or CDDP at different concentrations (0.25, 0.5 and 1 μM) and the total cellular and DNA-bound platinum contents were quantified using GF–AAS analysis (Figure 3).

The cellular internalization of **1** was dose-dependent and significantly higher than that of CDDP. In particular, at all tested concentrations, colon cancer cells treated with **1** showed a Pt content roughly ten times higher than that of CDDP-treated cells.

Similarly, concerning the DNA-platination levels, cells treated with **1** showed a higher content of Pt bound to DNA compared to those treated with CDDP. Surprisingly, the degree of Pt bound to DNA did not increase by increasing the compound concentrations, thus suggesting that the platination ability of **1** is not dose-dependent. This result highlights that the higher toxicity of **1** compared to CDDP could be ascribable to the combined effect of platinum-scaffold and chlorambucil on DNA rather than a result of an increase in the DNA platination ability of **1,** thus suggesting that chlorambucil plays a major role in the cytotoxicity of **1**.

Overall, these results clearly suggest that the conjugation of chlorambucil to Pt(IV) cisplatin-based scaffold has an impact on the lipophilic character of the prodrug **1,** as previously observed for analogue mono-substituted Pt(IV) species [16], thus inducing a substantial increase in cellular uptake and improving the DNA-targeting ability.

### 2.5. DNA Fragmentation and Apoptosis Induction

DNA fragmentation is a fundamental step in the apoptosis process, which follows the activation of endogenous endonucleases, in turn determining the cleavage of chromatin DNA into internucleosomal fragments.

**Figure 4:**
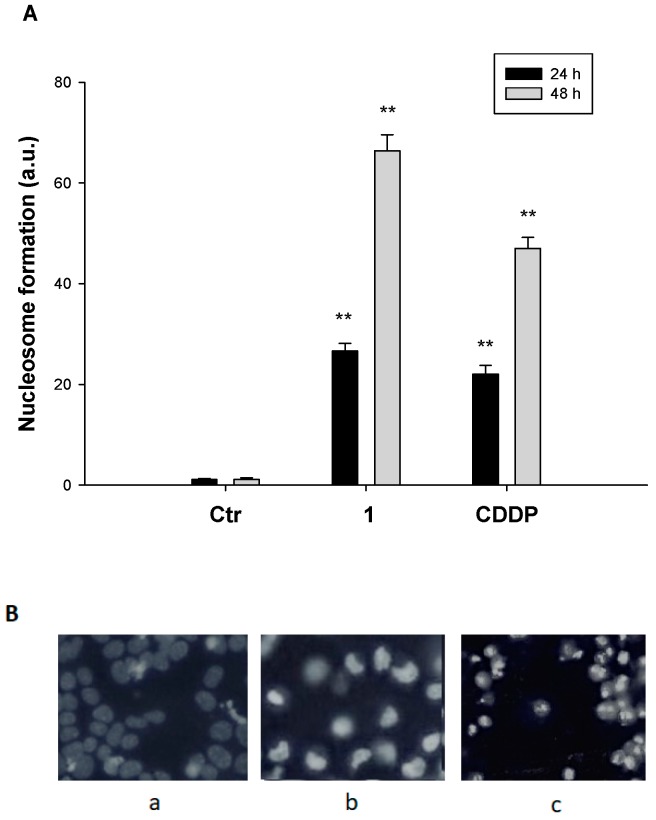
DNA fragmentation and apoptosis induction. (**A**) Nuclear DNA fragmentation. HCT-15 cells were treated for 24 or 48 h with IC_50_ of **1** or CDDP. Quantitative estimation of DNA fragmentation was obtained with an ELISA test. Error bars indicate SD. ** *p* < 0.01. (**B**) HCT-15 cells were treated with IC_50_ of **1** or CDDP for 48 h and stained with the fluorescent dye Hoechst 33258. (**a**) control cells; (**b**) CDDP-treated cells; (**c**) **1**-treated cells.

Following the treatment of HCT-15 cells with **1** for 24 or 48 h, a substantial increase in cells undergoing nuclear DNA fragmentation and apoptosome formation was detected (Figure 4A). Notably, following 48 h of treatment with **1**, a roughly 60-fold increase in nucleosome formation was detected in treated cells with respect to controls.

Apoptotic cell death induction was also detected through Hoechst 33342 staining. As depicted in Figure 4B, HCT-15 cells treated with **1** displayed pyknotic nuclei, chromatin condensation, and fragmentation characteristics, which are typical features of apoptosis induction.

## 3. Materials and Methods

Chlorambucil was purchased from TCI Europe and used as supplied. K_2_PtCl_4_, NHS (Hydroxysuccinimide), DCC (N,N′-Dicyclohexylcarbodiimide) and K_2_CO_3_ were purchased from Sigma-Aldrich. Cisplatin and Oxoplatin were synthesized as previously described [35,36].

All the other chemicals and solvents are of analytical or spectroscopic grade, and were obtained from commercial sources and used without additional purification. Deuterated solvents were obtained from Apollo Scientific.

The elemental analysis studies (carbon, hydrogen, and nitrogen) were performed by means of a PerkinElmer 2400 series II analyzer.

ESI Mass Spectra were recorded with a Waters LCT Premier XE Spectrometer.

NMR: ^1^H and ^13^C spectra were obtained in a solution of CDCl_3_ or DMSO-*d*_6_ and ^195^Pt NMR spectrum was obtained in a dimethylformamide (DMF) solution (Insert of D_2_O) at 300 K, in 5-mm sample tubes, with a premium shielded Agilent Varian 500 MHz (operating at 500.13, 125.75, and 107.49 MHz, respectively). The ^1^H chemical shift was referenced to the residual impurity of the solvent. The external reference was Na_2_PtCl_4_ in D_2_O (adjusted to δ = −1628 ppm from Na_2_PtCl_6_) for ^195^Pt.

HPLC studies: The stability and reduction **1** with ascorbic acid were followed using high-performance liquid chromatography (HPLC) with a Phenomenex Luna C18 (5 μM, 100 Å, 250 mm × 4.60 mm i.d.) column at room temperature at a flow rate of 1.0 mL/min with 254 nm UV detection. Mobile phase containing 80:20 acetonitrile (0.1% trifluoroacetic acid): water (0.1% trifluoroacetic acid): **1** was dissolved in DMF (0.5 ml) and diluted to a final concentration of 0.5 mM using acetonitrile and water solution (1/1) with 5 mM ascorbic acid and 2 mM 4-(2-hydroxyethyl)piperazine-1-ethanesulfonic acid (HEPES) buffer (pH 7). The process was followed at 37 °C until complete reduction. The HPLC chromatogram from the reduction reaction was compared with the chromatogram of pure samples of the tranilast.

### 3.1. Synthesis

Chlorambucil-NHS ester. Chlorambucil (0.25 g, 0.82 mmol) and NHS hydroxysuccinimide (0.095 g, 0.82 mmol) were dissolved in 25 mL of dry CH_3_CN and DCC, dissolved in dry CH_3_CN (10 mL, 122 mM), was added dropwise. A white solid immediately precipitated and the reaction mixture was stirred at RT overnight. The white solid obtained was filtered off and the yellow solution was dried under vacuum to give a yellow solid that was re-dissolved in 20 mL of dichloromethane and kept at 4^o^C overnight. Another small portion of white solid was formed and removed using filtration. The yellow solution was washed with 20 mL of 4% NaHCO_3_, dried over Na_2_SO_4_ and dried under vacuum, obtaining the product as a white solid (0.311 g, 94 %). Anal. Calcd. for C_18_H_22_Cl_2_N_2_O_4_: C 53.88, H 5.53, N: 6.98, found C 53.48, H 5.89, N 6.66. ^1^H NMR (500 MHz, CDCl_3_): δ = 7.01 (d, 2H), 6.56 (d, 2H), 3.62 (t, 4H), 3.55 (t, 4H), 2.77 (s, 4H), 2.57 (t, 2H), 2.52 (t, 2H), 1.95 (m, 2H). ^13^C NMR (125 MHz, CDCl_3_): δ 168, 167, 143, 128(2), 111, 52, 39, 32, 29, 25, and 24 ppm.

(**1**). Oxoplatin (0.1 g, 0.3 mmol) and chlorambucil-NHS ester (0.11 g, 0.27 mmol) were suspended in 5 mL of dry DMSO and the mixture was stirred at 60°C for 15 h. The yellow residue (oxoplatin in excess) was filtered off and the yellow solution was lyophilized overnight, obtaining a sticky yellow solid. The solid was dissolved in 4 mL of DMF and precipitated through the addition of Et_2_O. The solid was washed 3 times with 5 mL of dichloromethane and 2 times with 5 mL of Et_2_O and dried under vacuum overnight, giving **1** as a yellow solid (0.063 g, 0.10 mmol, 43%). Anal. Calcd. for C_14_H_25_Cl_4_N_3_O_3_Pt: C 27.11, H 4.06, N: 6.77, found C 27.64, H 3.85, N 6.36. ^1^H NMR (500 MHz, DMSO-*d*_6_): δ = 6.99 (d, 2H), 6.61 (d, 2H), 5.93 (m, 6H), 3.66 (m, 8H), 2.48 (t, 2H), 2.11 (t, 2H), 1.61 (t, 2H) ppm; ^13^C NMR (DMSO-*d*_6_, 125.75 MHz) δ 175, 145, 130, 129, 112, 53, 42, 35, 34, and 27 ppm; ^195^Pt NMR (107.49 MHz, DMF insert D_2_O): δ = 994 ppm. ESI-MS (-) peak at 619.02 m/z corresponding to (M-H)^−^.

### 3.2. Biological Methods

The platinum(IV) complex and the reference drug chlorambucil were dissolved in DMSO to stock solutions of 1 mg/mL just before the experiment, and the calculated drug amounts were added to the cell growth medium (final solvent concentration of 0.5%, which had no evident effect on cell killing). Cisplatin (CDDP) was dissolved in a 0.9% NaCl solution just before the experiments. CDDP, chlorambucil, and MTT (3-(4,5-dimethylthiazol-2-yl)-2,5-diphenyltetrazolium bromide) were obtained from Sigma Chemical Co., St.Louis, MI, USA.

Cell cultures: Human pancreatic (PSN-1 and BxPC3) and colon (HCT-15 and LoVo) carcinoma cells were obtained from American Type Culture Collection (ATCC, Rockville, MD, USA). The human thyroid carcinoma cell line (BCPAP) was provided by the Leibniz-Institut DSMZ—Deutsche Sammlung von Mikroorganismen und Zellkulturen GmbH (Braunschweig, Germany). The cisplatin-sensitive human ovarian cancer cell line (2008) and its cisplatin-resistant variant (C13*) were kindly provided by Prof. G. Marverti (Dept. of Department of Biomedical, Metabolic and Neural Sciences, University of Modena and Reggio Emilia, Italy). Human cervical (A431) carcinoma cells were kindly provided by Prof. F. Zunino (Istituto Nazionale per lo Studio e la Cura dei Tumori, Milan, Italy). Cell lines were maintained in the logarithmic phase at 37°C in a 5% carbon dioxide atmosphere using the following culture media containing 10% fetal calf serum (Euroclone, Milan, Italy), antibiotics (50 units·mL^−1^ penicillin and 50 µg·mL^−1^ streptomycin), and 2 mM l-glutamine: i) RPMI-1640 medium (Euroclone) for HCT-15, BCPAP, PSN-1, BxPC3, A431, 2008 and C13 cells; and ii) HAM’s F-12 for LoVo cells.

Spheroid culturing: Spheroids were obtained by seeding 2.5 × 10^3^ HCT-15 cells/well in a phenol red-free medium, containing 10% FCS and 20% methyl cellulose in round-bottom non-tissue culture treated 96 well-plates (Greiner Bio-one, Kremsmünster, Austria).

MTT assay: The cytotoxicity effect against human cancer cell lines was evaluated using MTT (tetrazolium salt reduction) assay. Briefly, 3–8 × 10^3^ cells/well, dependent on the growth characteristics of the cell line, were seeded in flat-bottom tissue culture treated 96 well microplates in a growth medium (100 µL), and then incubated at 37°C in a 5% carbon dioxide atmosphere. After 24 h, the medium was replaced with a fresh one containing the tested compounds at the appropriate concentration. Following 72 h of treatment, each well was treated with 10 µL of a 5 mg·mL^−1^ MTT saline solution, and after an additional 5 h, 100 µL of a sodium dodecylsulfate (SDS) solution in HCl 0.01 M was added. After an overnight incubation, the cell growth inhibition was detected by measuring the absorbance of each well at 570 nm, using the Bio-Rad 680 microplate reader (Bio-Rad, Hercules, CA, USA). The mean absorbance for each drug dose was expressed as a percentage of the control untreated well absorbance and plotted vs the drug concentration. IC_50_ values represent the drug concentrations that reduced the mean absorbance at 570 nm to 50% of those in the untreated control wells.

Acid phosphatase (APH) assay: An APH modified assay was used for determining cell viability in 3D spheroid systems. Briefly, the pre-seeded spheroids were treated with a fresh medium containing the compound to be studied at the appropriate concentration. After 72 h, each well was treated with 100 μL of the assay buffer (0.1 M sodium acetate, 0.1% Triton-X-100, supplemented with ImmunoPure p-nitrophenyl phosphate; Sigma Chemical Co., St.Louis, MI, USA) and, following an additional 3 h of incubation, 10 μL of 1 M NaOH solution was added. Cell growth inhibition was detected by measuring the absorbance of each well at 405 nm, using a Bio-Rad 680 microplate reader. The mean absorbance for each drug dose was expressed as a percentage of the control untreated well absorbance (T/C) and plotted vs drug concentration. IC_50_ values, the drug concentrations that reduce the mean absorbance at 405 nm 50% of those in the untreated control wells, were calculated using a four-parameter logistic (4-PL) model. The evaluation was based on means from at least four independent experiments.

Cellular uptake: HCT-15 cells (2.5 × 10^6^) were seeded in 75 cm^2^ flasks in a growth medium (20 mL). After 24h incubation, the medium was replaced and the cells were treated with tested compounds for 24 h. The cells were then washed twice with cold PBS, harvested, and counted. Samples were subjected to three freezing/thawing cycles at -80 °C, and then vigorously vortexed. The samples were added with highly pure nitric acid (Pt: ≤0.01 μg·kg^−1^, TraceSELECT^®^ Ultra, Sigma Chemical Co.) and mineralized by using a speed wave MWS-3 Berghof instrument (Eningen, Germany). Each mineralized sample was analyzed by using a Varian AA Duo graphite furnace atomic absorption spectrometer (Varian, Palo Alto, CA; USA) to detect the platinum content at 324.7 nm. Calibration curves were performed by using standard solutions (Sigma Chemical Co., St.Louis, MI, USA).

DNA platination studies: HCT-15 cells (about 6 × 10^6^) were treated with tested complexes for 24 h. DNA was extracted and purified using a commercial spin column quantification kit (Qiagen DNeasy Blood and Tissue Kit, Venlo, The Netherlands). Highly purified samples (A260/A230 > 1.8 and A260/A280 > 2.0) were dried and re-dissolved in 200 µL of Milli-Q water (18.2 MΏ) at 65 °C in a shaking thermo-mixer, and subsequently mineralized and analyzed for total platinum content using GF–AAS, as described above.

Nuclear DNA fragmentation: HCT-15 cells (10^4^/well) were treated in a 96 well plate at 37 °C for 24 and 48 h with IC_50_ doses of tested compounds. Afterwards, the plate was centrifuged for 10 min, the supernatant removed, and the pellet treated according to the manufacturer’ instructions for the ELISAplus cell death detection kit (Roche, Basel, Switzerland). The extent of nuclear fragmentation was measured in a plate reader following absorbance at 405 minus 492 nm using a fluorescence microplate reader (Fluoroskan Ascent FL, Labsystem, Finland).

Hoechst 33258 staining: HCT-15 cells were seeded into 8-well tissue-culture slides (BD Falcon, Bedford, MA, USA) at 5 × 10^4^ cells/well (0.8 cm^2^). After 24 h, the cells were washed twice with PBS and, following 48 h of treatment with IC_50_ doses of the tested compound, cells were stained for 5 min with 10 μg/mL of Hoechst 33258 (2′-(4-hydroxyphenyl)-5-(4-methyl-1-piperazinyl)-2,5′-bi-1H-benzimidazole trihydrochloride hydrate, Sigma-Aldrich, St.Louis, MI, USA) in PBS before being examined using fluorescence microscopy (Olympus, Tokyo, Japan).

Statistical analysis: All values are the means ± SD of no less than three measurements, starting from three different cell cultures. Multiple comparisons were made using ANOVA followed by the Tukey–Kramer multiple comparison test (** *p* < 0.01) using GraphPad Prism 5.03 for Windows software (GraphPad Software, La Jolla, CA, USA).

## 4. Conclusions

In this study, a Pt(IV) prodrug **1** combining chlorambucil and cisplatin was prepared and thoroughly characterized by means of spectroscopic and spectrometric techniques. Tested on a panel of several human cancer cell lines and compared to that of the precursors CDDP and chlorambucil, compound **1** proved to be much more cytotoxic than the reference compounds, being significantly effective also against human cancer cells endowed with scarce sensitivity or resistant to CDDP. The antitumor potential of Pt(IV) derivative **1** was also confirmed in 3D colon tumor cells, and in this spheroid cancer cell model **1** was shown to be comparable with that of OXP. 

Uptake studies in colon cancer cells confirmed that the conjugation of chlorambucil to Pt(IV) cisplatin-based scaffold tunes the lipophilicity of the prodrug, consequently improving the ability of the compound to cross cell membrane and to accumulate into cancer cells. In addition, DNA-platination and apoptosis induction studies confirmed that **1** does exert its antitumor activity by covalently binding to DNA, leading to the activation of apoptotic cancer cell death. However, the DNA-targeting ability of **1** was significantly higher than that showed by the reference Pt(II) drug, CDDP. Overall, our results clearly point out the potential of combining two DNA-recognizing molecules in the same chemical entity for the development of target-specific anticancer agents.

## Figures and Tables

**Table 1:** Cytotoxicity against human cancer cell lines.

Compound	IC_50_ (µM) ± SD							
	PSN1	2008	C13*	LoVo	A431	BxPC3	HCT-15	BCPAP
1	0.42 ± 0.1	0.31 ± 0.1	0.28 ± 0.1	0.98 ± 0.1	0.41 ± 0.1	1.74 ± 0.5	0.39 ± 0.1	0.51 ± 0.2
Chlorambucil	>100	34.58 ± 16.5	>100	>100	74.48 ± 8.2	>100	44.52 ± 9.5	>100
CDDP	18.11 ± 3.2	2.18 ± 0.9	22.11 ± 3.2	9.15 ± 2.1	2.21 ± 0.4	7.22 ± 2.2	15.28 ± 2.6	7.31 ± 1.2

CDDP: cisplatin, SD = standard deviation. Cells (3–5 × 10^4^·mL^−1^) were treated for 72 h with different concentrations of tested compounds. Cytotoxicity was assessed using the MTT test. IC_50_ values were calculated using a four-parameter logistic model (*p* < 0.05).

**Table 2:** Cytotoxicity towards human HCT-15 cancer cell spheroids.

	IC_50_ (µM) ± SD		
	1	CDDP	OXP
HCT-15	40.4 ± 5.1	71.1 ± 3.9	33.2 ± 4.6

Spheroids (3 × 10^3^ cells/well) were treated for 72 h with tested compounds and the growth inhibitory activity was assessed using APH assay. IC_50_ values were calculated from the dose-survival curves using the four-parameter logistic model (*p* < 0.05). SD = standard deviation.

## Supplementary Materials

Supplementary materials can be found at http://www.mdpi.com/1422-0067/19/12/3775/s1.
