# Supplementary material for: A Pt(IV) Prodrug Combining Chlorambucil and Cisplatin: A Dual-Acting Weapon for Targeting DNA in Cancer Cells
Source: Int J Mol Sci. 2018 Nov 27;19(12):3775. doi: 10.3390/ijms19123775 (PMC6321036; doi:10.3390/ijms19123775)
Supplement: Supplementary file 1 [file ijms-19-03775-s001.pdf]

A Pt(IV) Prodrug Combining Chlorambucil and Cisplatin: a Dual-Acting Weapon for Targeting DNA in Cancer Cells

Diego Montagner [1,*], Dina Tolan [2,3], Emma Andriollo [4], Valentina Gandin [4,*] and Cristina Marzano [4]

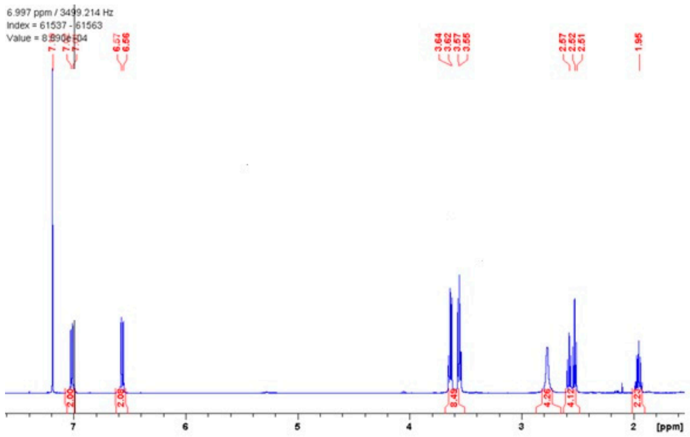

Figure S1. ^{1}H NMR of Chlorambucil-NHS Ester in $CDCl_3$.

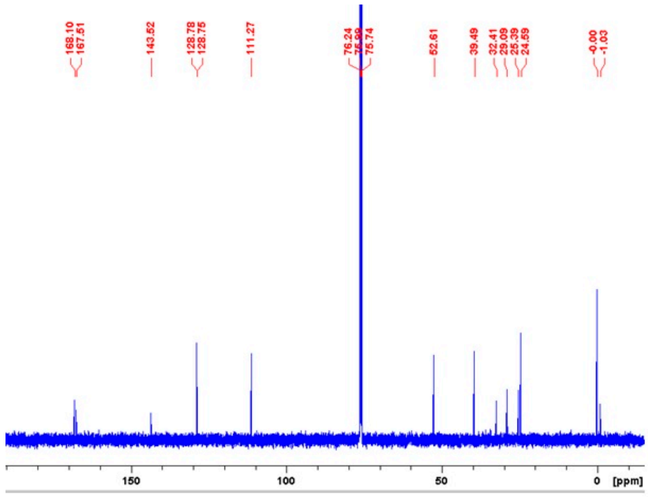

Figure S2. ^{13}C NMR of Chlorambucil-NHS Ester in $CDCl_3$.

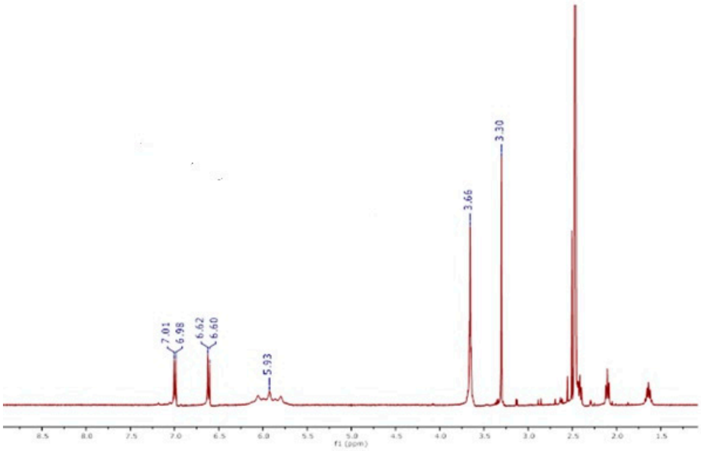

Figure S3. ^{1}H NMR of **1** in DMSO-*d*$_{6}$.

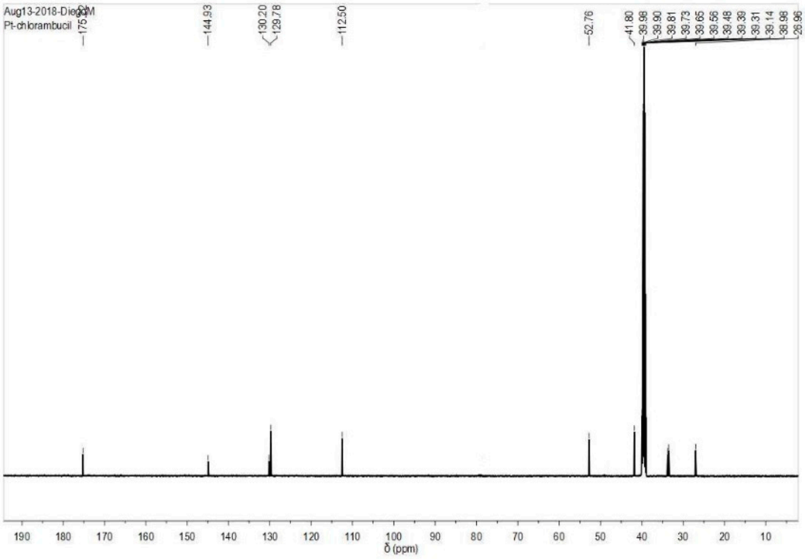

Figure S4. ^{13}C NMR of **1** in DMSO-*d*$_{6}$.

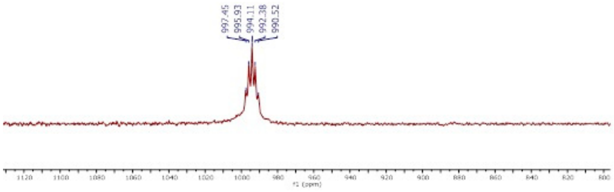

Figure S5. ^{195}Pt NMR of **1** in DMF (D_2O).

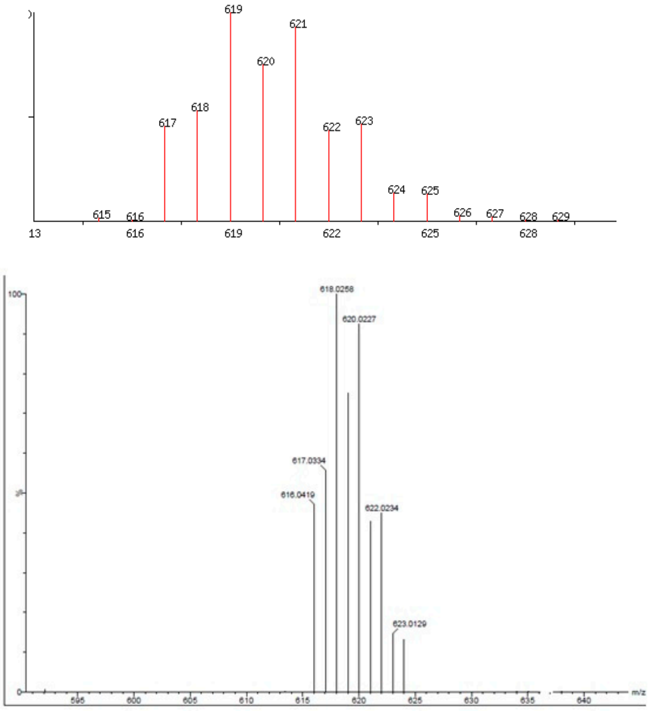

Figure S6. Top: simulated ESI Mass spectrum of **1** (-); bottom: ESI Mass spectrum of **1** (-).
